# Developing Core Indicators for Evaluating Second Victim Programs: An International Consensus Approach

**DOI:** 10.3389/ijph.2024.1607428

**Published:** 2024-08-30

**Authors:** Sofia Guerra-Paiva, Irene Carrillo, José Mira, Joana Fernandes, Reinhard Strametz, Eva Gil-Hernández, Paulo Sousa

**Affiliations:** ^1^ Public Health Research Centre, NOVA National School of Public Health, NOVA University of Lisbon, Lisboa, Portugal; ^2^ Comprehensive Health Research Center, NOVA National School of Public Health, NOVA University of Lisbon, Lisboa, Portugal; ^3^ Department of Health Psychology, Miguel Hernández University, Elche, Spain; ^4^ Fundación para el Fomento de la Investigación Sanitaria y Biomédica de la Comunitat Valenciana (FISABIO), Valencia, Spain; ^5^ NOVA National School of Public Health, NOVA University of Lisbon, Lisbon, Lisboa, Portugal; ^6^ Wiesbaden Business School, RheinMain University of Applied Sciences, Wiesbaden, Germany

**Keywords:** patient safety, second victim, programs, evaluation, indicators

## Abstract

**Objectives:**

To establish a consensus for evaluating second victims (SV) support interventions to facilitate comparison over time and across different organizations.

**Methods:**

A three-phase qualitative study was conducted from June 2023 to March 2024. This consensus approach engaged members of the European Researchers Network Working on Second Victims. A nominal group technique and insights from a scoping review were used to create a questionnaire for Delphi Rounds. Indicators were rated 1–5, aiming for agreement if over 70% of participants rated an indicator as feasible and sensitive with scores above 4, followed by a consensus conference.

**Results:**

From an initial set of 113 indicators, 59 were assessed online, with 35 advancing to the Delphi rounds. Two Delphi rounds were conducted, achieving response rates of over 60% and 80% respectively, resulting in consensus on 11 indicators for evaluating SV support programs. These indicators encompass awareness and activation, outcomes of SV support programs, as well as training offered by the institution.

**Conclusion:**

This study presents a scoreboard for designing and monitoring SV support programs, as well as measuring standardized outcomes in future research.

## Introduction

Healthcare workers (HCWs) frequently encounter potential traumatizing events resulting from the process of care within healthcare settings. These type of events have been associated with unexpected patient harm or death resulting from the care process (adverse events) [1, 2]. Evidence suggest that this type of events affect 1 in 10 patients [3].

Moreover, healthcare incidents that create potential risks to the system without directly affecting patient wellbeing (near misses) can still have psychological and emotional impacts on HCWs [4].

In 2000, Albert Wu introduced the term “second victim” (SV) to describe healthcare providers who experience emotional distress, guilt, or trauma following an adverse event involving a patient, such as a medical error or an unexpected patient outcome [5]. Recently, the European Researchers’ Network Working on Second Victims (ERNST) has refined the SV definition to include “any healthcare worker, directly or indirectly involved in an unanticipated adverse patient event, unintentional healthcare error, or patient injury, and who becomes victimized in the sense that they are also negatively impacted” [6].

It is well established that 60%–92% of HCWs experience the Second Victim Phenomenon (SVP) at least once in their careers [1, 7–13]. This phenomenon can have significant medium to long-term psychological and physical effects that impact their professional and personal lives [2, 7, 8, 10].

While this phenomenon is not new, health authorities increasingly recognizing the importance of providing support to HCWs following highly stressful events and enhancing the psychological safety within healthcare organizations.

Evidence refers that supporting HCWs after a stressful event improves patient safety [14, 15] and reduces avoidable costs [4]. Thus the importance of creating psychological safe environments, by strengthening the sense of safety to take interpersonal risks at work [16], will encourage supportive interactions such as SV programs [17].

In recent years there has been growing investment in SV support programs worldwide. Most of these programs have been developed in hospital settings, tailored to respond to healthcare needs following stressful events [18]. Most of the implemented programs are based on peer support, with the primary goals of increasing HCWs’ wellbeing, decreasing their emotional stress in work and ensuring patient safety [19, 20].

The support programs vary in format, including the use of hotlines, individual and groups sessions [18]. The ForYOU [21] and the RISE [22] programs were the pioneers in this field and have been adopted in various institutions across Europe [23, 24]. They have demonstrated that peer support is the most accepted and desired method for aiding SVs in healthcare [25].

The majority of published information regarding SV support programs in Europe and beyond indicates that they were successfully implemented [18]. However there is still a gap regarding the evaluation process of these types of interventions, particularly over longer periods [18].

A scoping review identified and organized the indicators used to evaluate SV support programs in five main categories: outcomes related to support services utilization, evaluation of the program by the peer supporters perspective, evaluation of the program by the user perspective (HCWs involved in PSIs/SV), health-related and work-related outcomes [18]. However, there is no consensus on the most appropriate indicators for evaluating SV programs or on which indicators should be prioritized. Reaching an agreement on the most suitable indicators to measure this type of intervention is urgent. Such an agreement could facilitate the comparison of SV programs and guide the implementation and adjustment of future interventions.

The regular evaluation (monitorization) involving the application of appropriate indicators in a timely manner is highly useful for healthcare organization [26]. Evaluating programs provides crucial information that helps decision-makers and health organizations understand the impact of healthcare interventions and make informed decisions [26–28]. In this sense, the indicator should serve an intended function that supports a decision-making (“*fit for purpose*”) and it should deliver the information to the “right” place at the right time (“fit for use”) [29]. Moreover, indicators allow for adjustments based on the needs of the services, and enable comparison of observed outcomes [27].

The criteria for selecting indicators highly depends on the purpose, type of sources, the availability of the data, and its practical value [26]. This is closely related to the feasibility of the indicator. Evidence has been shown the importance of creating feasible indicators which refers to the facility with which the quality indicator can be measured in accurate way [30]. This is highly related with their validity, which refers to the true condition of the event being measured [30, 31] and reliability (“the level of reproducibility and consistency between two or more measurements”) [32]. Also the indicators should be sensitive (how well a test can classify subjects who truly have the condition of interest [33]).

Given the widespread adoption of SV’ support programs, it seems urgent to agree on a minimal set of indicators to assess them over-time. The creation of these indicators should be guided by evidence-based information combined with clinical expertise and in some cases incorporating patient perspectives [34].

Considering the various organizational models and social aspects related to the conceptualization of human fallibility across countries, it is advisable to developed this set of indicators from an international perspective [35].

### Purpose of the Study

The aim of this study is to establish a consensus of indicators to evaluate the SV support interventions in order to facilitate their comparison over time and with other healthcare services. This will enable to define a minimal set of indicators to prove the useful application of SV support interventions in healthcare organizations, to ensure their rigor and evaluate their quality.

## Methods

A three-phase qualitative study was conducted from June 2023 to March 2024 to define an international consensus on a set of indicators to evaluate SV support programs. A Consensus Executive Board (CEB) was created to make decisions at each stage of the study. The CEB was created by four researchers (JM, PS, IC, SGP), all of whom had background in health management, quality improvement, patient safety, and SVP.

The Phase 0 brought insights gained from a scoping review, providing evidence-based starting point for the study. The scoping review aimed to identify the key factors for the effective implementation of SV support programs, including the metrics necessary to measure this type of interventions. In this phase, a collection of indicators that had been used was gathered.

Following Phase 0, in the phase 1 a nominal group technique was applied. This group, comprising invited experts from the ERNST (COST Action 19113), was tasked with generating and prioritizing a set of indicators based on their validity and reliability. Their empirical knowledge and expertise facilitated the construction of Questionnaire 0 composed by the final list of indicators to be scored in the next phase.

In Phase 2, the Delphi technique was applied. The goal here was to reach a consensus on the most feasible and sensitive indicators for evaluating SV programs. This consensus was based on the set of indicators defined in the earlier phases of the study.

The methodology for this study was guided by the recommendations of Nasa, Jain, and Juneja for Delphi studies, as well as the Standards for Reporting Qualitative Research (SRQR).

### Population of the Study and Sources of Information

The study population consisted of a multicultural group of ERNST members, representing organizations at different stages of addressing the SVP, spanning from early stages to those already seasoned in SV support programs.

The sources of information included the results of a published scoping review [18], designed phase 0 of the study. The scoping review encompasses a comprehensive search in nine different databases (CINAHL, Cochrane Library, Embase, Epistemonikos, PsycINFO, PubMed, SciELO Citation Index, Scopus, Web of Science Core Collection). Relevant websites were consulted, and reference lists of the studies included in the full-text screening were checked to identify any other potential articles to include. The search strategy applied in the scoping review is described in Supplementary Table 1. The search did not restrict the period of time or language of the included studies to ensure the most comprehensive overview of the existing literature and to reduce the selection bias. Editorials, letters to the editor, case series, case reports, narrative reviews and commentaries were excluded in this study.

In phase 1 of this study, we invited academics and research experts actively involved in the ERNST activities, and other suggested ERNST members with deep knowledge on quality improvement, patient safety and SVP to participate in the nominal group. All the participants had a clinical and academic background and research profile. This group was responsible to generate new indicators, complementing the collected data from the literature.

In the phase 2 of the study, the participant scope was expanded to encompass a wider array of experts, including HCWs, researchers, managers, and academics. In total, 81 individuals were invited to participate in the Delphi study. All of them had previously collaborated on SVP research, undergone intensive training, or possessed experience with SV support programs. All the members of Working groups 2 and 3 as well as Core group members of ERNST, were invited to participate in phase 2. All these members had previous experience in SV support programs or have been involved in research/training on SVP and patient safety. Additionally, we included some extra participants recommended by the initial group, who possessed expertise in SVP. In this study we aimed to achieve the gold standard of 60%–80% for survey response rates [36].

**FIGURE 1:**
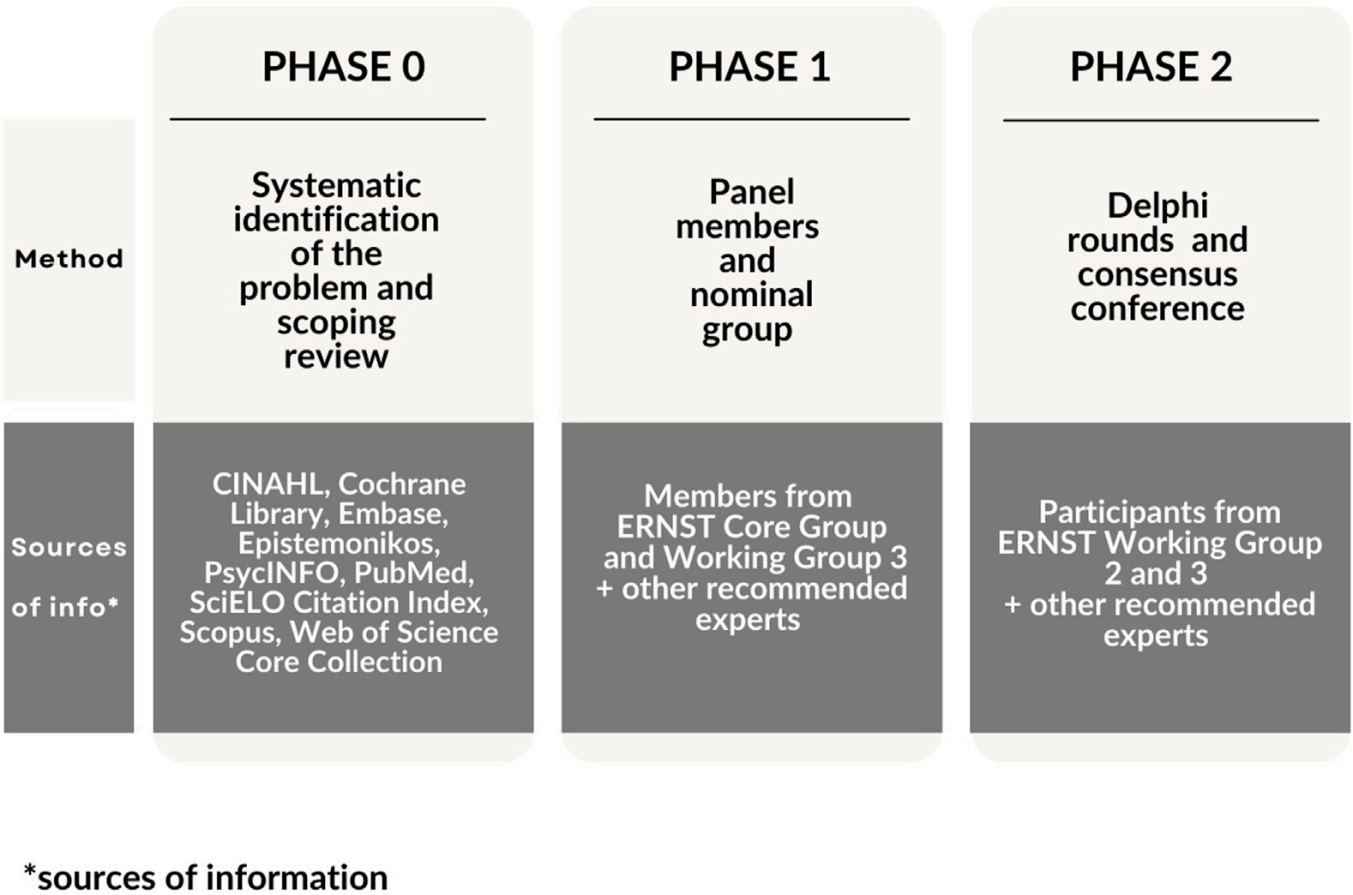
Phases of the study to find a consensus of second victim support interventions indicators (online and Lisbon, Portugal, 2024).

The phases of this study and detailed information about the sources of information and participants of each phase to define a set of indicators to evaluate SV programs are depicted in Figure 1.

In the following sections we will describe the different phases of the study in more detail.

### Phase 0 – Identification of the Problem

Prior to the qualitative study, the research team conducted a scoping review that collected metrics used to evaluate SV programs elsewhere. We used this information to complement the collected data retrieved from the phase 1 and 2 with previous evidence-based information. This is the reason why we defined this phase as point 0.

The scoping review was focused on a comprehensive understanding what existing organizational factors, relevant actors, contextual factors, operational attributes are present in interventions successfully implemented in health organizations to support second victims. The Joanna Briggs Institute [37] criteria were employed to conduct the scoping review.

In this study, 9,708 records were retrieved from the 9 databases, 43 articles were retrieved from the reference lists of the included articles, 11 from websites and 4 were collected from stakeholders’ group inputs. The detailed information of data collection, screening process, duplicates removed and reasons for exclusion is exhibited in the flow chart in line with the original Preferred Reporting Items for Systematic Reviews and Meta-Analyses (PRISMA) statement. This can be consulted in the Supplementary Table 2.

Since our aim was focused on collecting the metrics to evaluate SV programs, we only focused on the section “Organizational factors” of the scoping review.

### Phase 1 – Definition of a List of Indicators

In the phase 1, a nominal group technique was applied during a 2-day hybrid meeting of ERNST that was held in Lisbon in June 2023.

**FIGURE 2:**
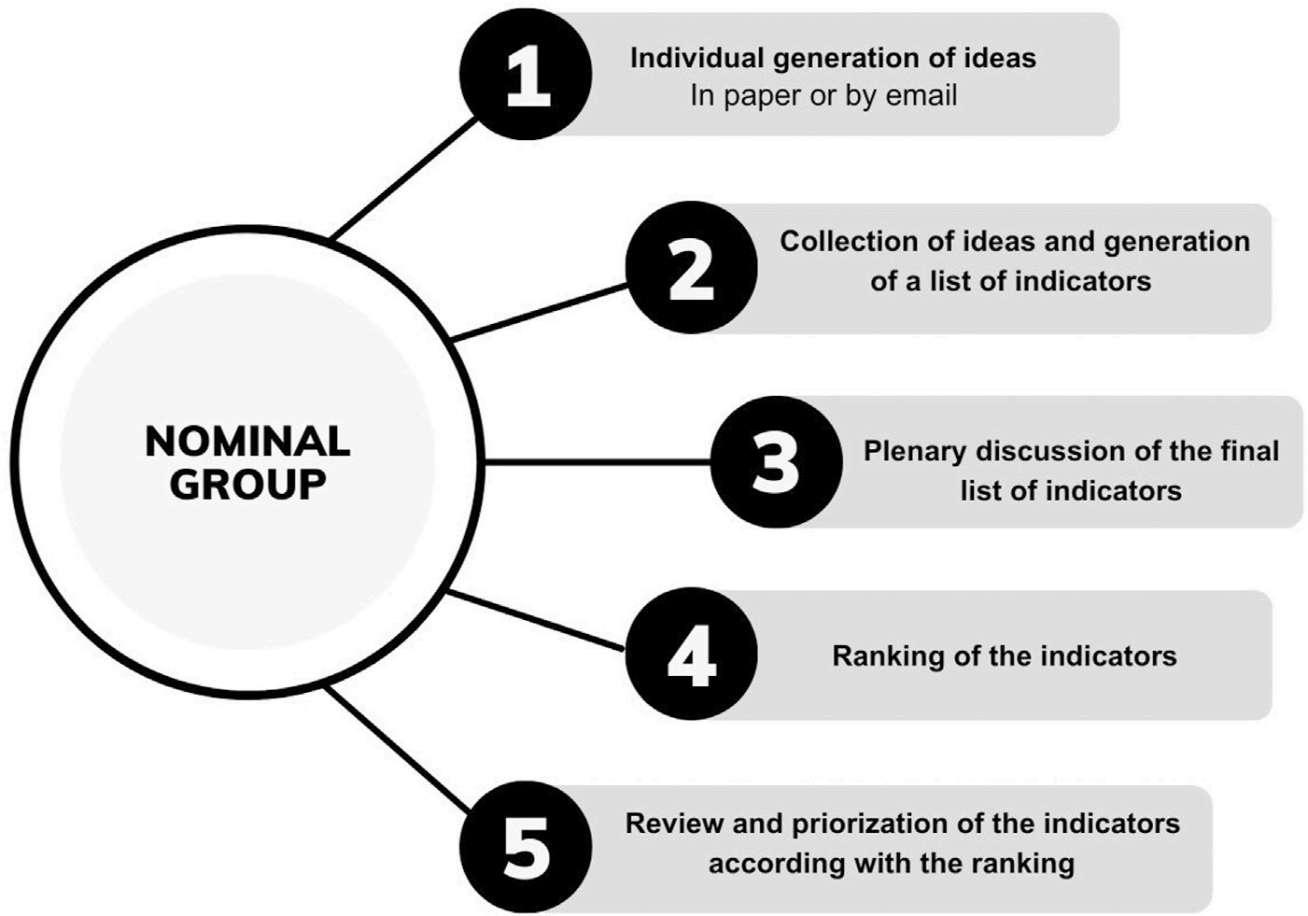
Stages of nominal group application (Lisbon, Portugal, 2024).

On the first day, the group focused on stages 1 and 2 of the method. The second session saw the application of stages 3, 4 and 5. All the five stages are detailed in Figure 2.

The conclusions of this phase led to the construction of questionnaire 0, incorporating a set of indicators to be ranked in phase 2 of the study using the Delphi technique.

#### Recruitment of Participants

Participants from the ERNST Consortium were invited based, firstly, on their experience in support interventions and, secondly, on their willingness to engage in the study. The ERNST members were prioritized to join the meeting, since it was funded by European Cooperation in Science and Technology (COST), in the scope of Cost Action 19113- ERNST.

Only ERNST members with clinical and academic profile along with previous research experience on quality improvement, patient safety and SVP were included in this phase of the study. The recruitment technique was based on the available information about the members of the ERNST consortium. We asked the leaders of the working groups and successful experiences in Europe that were part of the consortium if they would be willing to participate or if they could recommend someone from their teams to participate in this study.

The snowball sampling technique was employed to ensure that the experience and profile of the participants met the aims of the study. This is described as the process in which participants who are part of the study recommend at least one more potential ERNST member who meet the inclusion criteria and that were available to participate in the meeting. As each new members were added, they in turn suggest additional participants, allow in the sample to grow. This process was carried out consecutively as new members were added. In this way, the sample progressively increased [38].

#### Stage 1- Individual Generation of Ideas

Researchers agreed upon a script to facilitate this process, which included a main question: “What metrics are feasible and sensitive to evaluate SV support interventions?” This was supplemented by cluster questions targeting:

• hospital with previous experience on SV programs;
• hospital without previous experience on SV programs;
• non-hospital settings (primary care settings and long-term settings) in this type of interventions.

The metrics were required to be both valid (measuring what they are intended to) and reliable (providing consistent measurements across different populations and contexts). The participant were invited to suggest indicators based on the elements proposed in Donabedian’s model for measuring improvement and quality of care: structural, process, and outcome [39].

#### Stage 2 – Collection of the Ideas and Generation of a Final List of Indicators

The generated data was collected and organized by four researchers of the CEB (SGP, JF, IC, EGH) who reviewed the proposed indicators, identified similarities, and removed repetitions. A final list of indicators was defined, which included a group of indicators for further discussion and evaluation in subsequent stages of the nominal group.

#### Stage 3 – Plenary Discussion

The final list of indicators defined from stage 2 was discussed by all the participants of this phase in a plenary session. During this session, participants reviewed and potentially expanded the list of indicators.

#### Stage 4 – Ranking and Prioritization of the Indicators

The final list of indicators was ranked using the platform Quizizz [40]. Each indicator was evaluated individually and anonymously by all participants simultaneously using an electronic device. The evaluation employed a 5-point Likert scale ranging from hardly feasible, partially feasible, feasible, feasible and sensitive, and excellent for all settings.

#### Stage 5 – Review and Priorization of the Indicators

The evaluation of the indicators was coordinated online by IC and EGH. The collected data was organized in an Excel document, and the research team discussed the final group of indicators to be included in the Delphi technique.

### Phase 2 – Priority Setting and Analysis of Consensus

In this phase, a Delphi technique and a consensus conference were applied to score a group of indicators defined in the previous phases of this study.

The entire Delphi study was conducted from 20th September, 2023 to 27th March 2024. The first round ran from 20th September to 24th November 2023. During this period, two reminders were sent by email to the invited group of participants (10th October and 10th November). The second round was conducted from 12th December 2023 to 7th March 2024. In this round, three reminders were sent during this round (on 10th January, 15th February, and 27th February), with extended accounting for holiday breaks.

**FIGURE 3:**
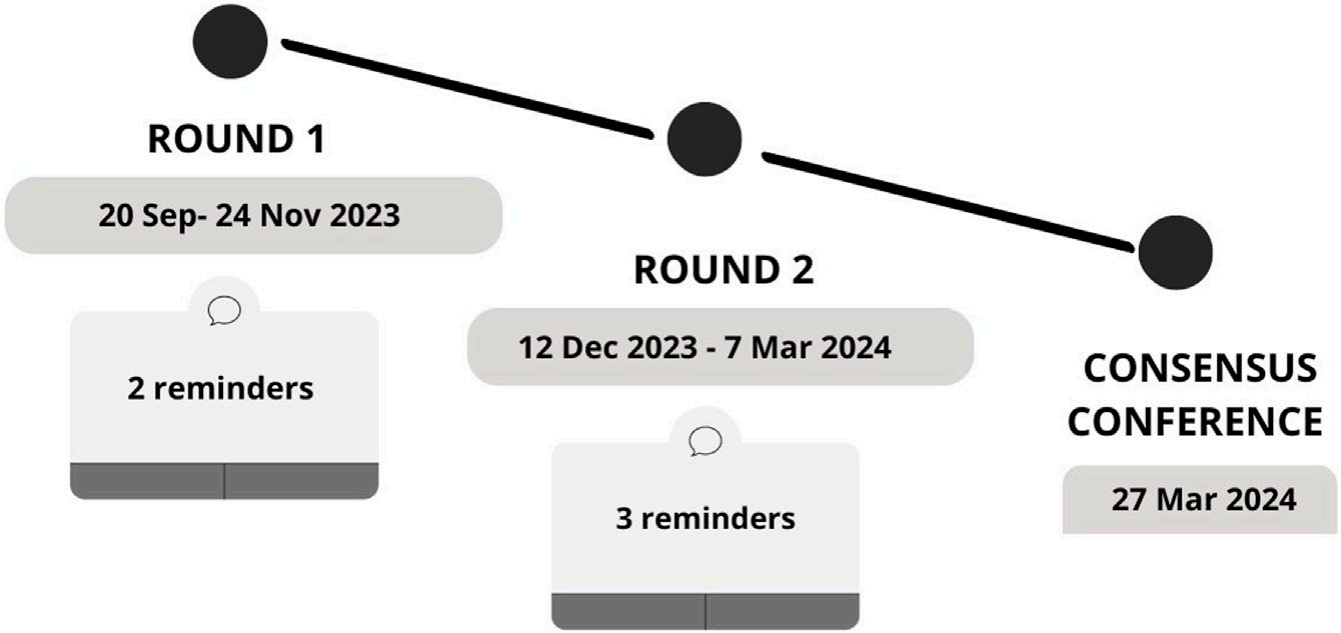
Timeline of the Delphi rounds (online, 2024).

The detailed timeline of the Delphi rounds and sent reminders is the illustrated in Figure 3.

The Delphi rounds were focused on scoring the 35 indicators defined in phase 1. The scoring enabled priority setting and guided the inclusion and exclusion of the indicators through the different Delphi rounds. The indicators were scored using an electronic platform, specifically costumized for conducting the Delphi Study. This platform was hosted on the secure servers of the Miguel Hernández University of Elche (Spain) (available in https://calite.umh.es/delphis/en/).

#### Application of the Delphi Technique

The application of the Delphi technique is characterized by anonymity, iteration, controlled feedback, and statistical aggregation of the group responses [41]. This method proves particularly effective in situations requiring priority setting [42].

As recommended by Nasa, Jain and Juneja [43], for the criteria for the panel included the homogeneity of the panel, labelling members as “experts,” and maintaining a panel size between 30 and 60 to reflect the diversity of European experiences.

We employed an online platform for individual and anonymous evaluation of the indicators included in the Delphi questionnaire.

The evaluation of the indicators was summarized in two main categories to decrease the level of burden of the questionnaire applied to the Delphi panel and avoid high dropout rates:

• The degree in which the indicator can be easily measured in an accurate way (feasibility) [30]: the proposed indicator is valid (can be measured and reflects the truth), reliable (“always in the same way”) and is pertinent to the objective of the measure (to evaluate interventions to support SVs).
• The extent to which the indicator accurately reflects changes in implementation [44] (sensitivity/responsiveness): the proposed indicator represents an improvement in the implementation process, performance, or results of an intervention supporting SVs.

In the first category, we have grouped three different components of quality measure (reliability, validity and pertinence) as these are essential elements to ensure the feasibility of the indicator (the degree in which the indicator can be easily measured in an accurate way).

In the second category, we pretend to analyze the extent in which the indicator can reflect the reality. We aimed to evaluate if the collected data is meaningful and effectively detects small changes during the measurement. This will ensure to collect the accurate data and will provide appropriate feedback to enhance the intervention over time.

The participants were notified via email to respond to an online survey on a scale from 1 to 5. In this scale the minimum score (rated as 1) indicated difficulty in measurement (not feasible)/low sensitivity to changes (not sensitive), and the highest score (rated as 5) indicated ease of measurement (very feasible)/high sensitivity to changes (very sensitive). The inclusion and exclusion of the indicators in the different rounds were determined by the mean scores of the total panel participants.

#### Round 1

In round 1, indicators scoring ≤3.5 in either feasibility or sensitivity during the initial round were excluded. Those scoring >3.5 but <4.0 in at least one of these domains were retained for further assessment in the subsequent round. The indicators that scored ≥4 were directly included to integrate the consensus conference.

#### Round 2

In round 2, participants had the option to adjust their scores based on the provided summary information or to retain their original evaluations. During the second round, indicators submitted for reassessment were accompanied by both the group’s average score and the individual participant’s score for each element from round 1. Additionally, participants could identify priority indicators by ticking a checkbox. For inclusion in the consensus conference discussion, indicators needed to be prioritized by more than 50% of the participants (n = 20) and achieve an agreement score of ≥4 from over 70% of the participants.

In summary the criteria applied in the second round were as follows:

- Considered a priority by more than 20 participants;
- Score >4 in feasibility and sensitivity by at least 70% of the participants.

After submitting the scores for each round, the collated data was automatically analyzed to determine a consensus on a set of indicators for evaluating SV programs. The final consensus was reached upon meeting a predefined minimum agreement on the indicators needed to assess these programs.

#### Consensus Conference

After completion of the Delphi rounds, the final results were deliberated upon by the CEB during a consensus conference. The aim of this conference was to achieve agreement among the research team regarding the selection of indicators for evaluating SV support programs. During the conference, the CEB reviewed the aggregated responses from the Delphi rounds, considered any divergent viewpoints, and engaged in thorough discussions to define the final list of indicators. This collaborative process ensured that the selected indicators reflected the collective agreement reached by the expert panel.

## Results

### Phase 0

A total of 22 indicators to evaluate SV support programs were selected by the CEB based on the scoping review data. The selection of the indicators was based on the discussion of what were the most adequate indicators to evaluate SV programs evaluation. These indicators were organized into nine main domains by the CEB members.

The collected indicators and respective literature are detailed in Supplementary Table 3.

### Phase 1

Fifteen participants took part in the nominal group, with eleven attending in person and four participating online using the Zoom platform. This was a multidisciplinary group from 12 different countries, all of whom were academics, clinicians, and researchers with previous background in health management and patient safety, actively working on SVP initiatives. Detailed information about the participants is available in Supplementary Table 4.

During the initial phase of the nominal group, 91 indicators were independently generated by the 15 participants, supplementing the 22 indicators gathered from the scoping review (phase 0). In the second stage two researchers synthesized and consolidated these into a final list of 59 items (the rating can be consulted in Supplementary Table 5). During the subsequent plenary session (stage 3), four indicators were excluded after thorough debate. After rating the indicators using an online platform (stage 4) and further CEB review (stage 5), a refined set of 35 indicators was selected for integration into the questionnaire 0. The 35 indicators were organized in four main domains:

- indicators related to the SV program (outcome indicators);
- indicators related to the intervention process and structure;
- indicators related to the SV experience the SV experience;
- indicators related to the healthcare organization and culture; This classification of the indicators contributed to organize the questionnaire 0 that was applied in the second phase of this study.

**FIGURE 4:**
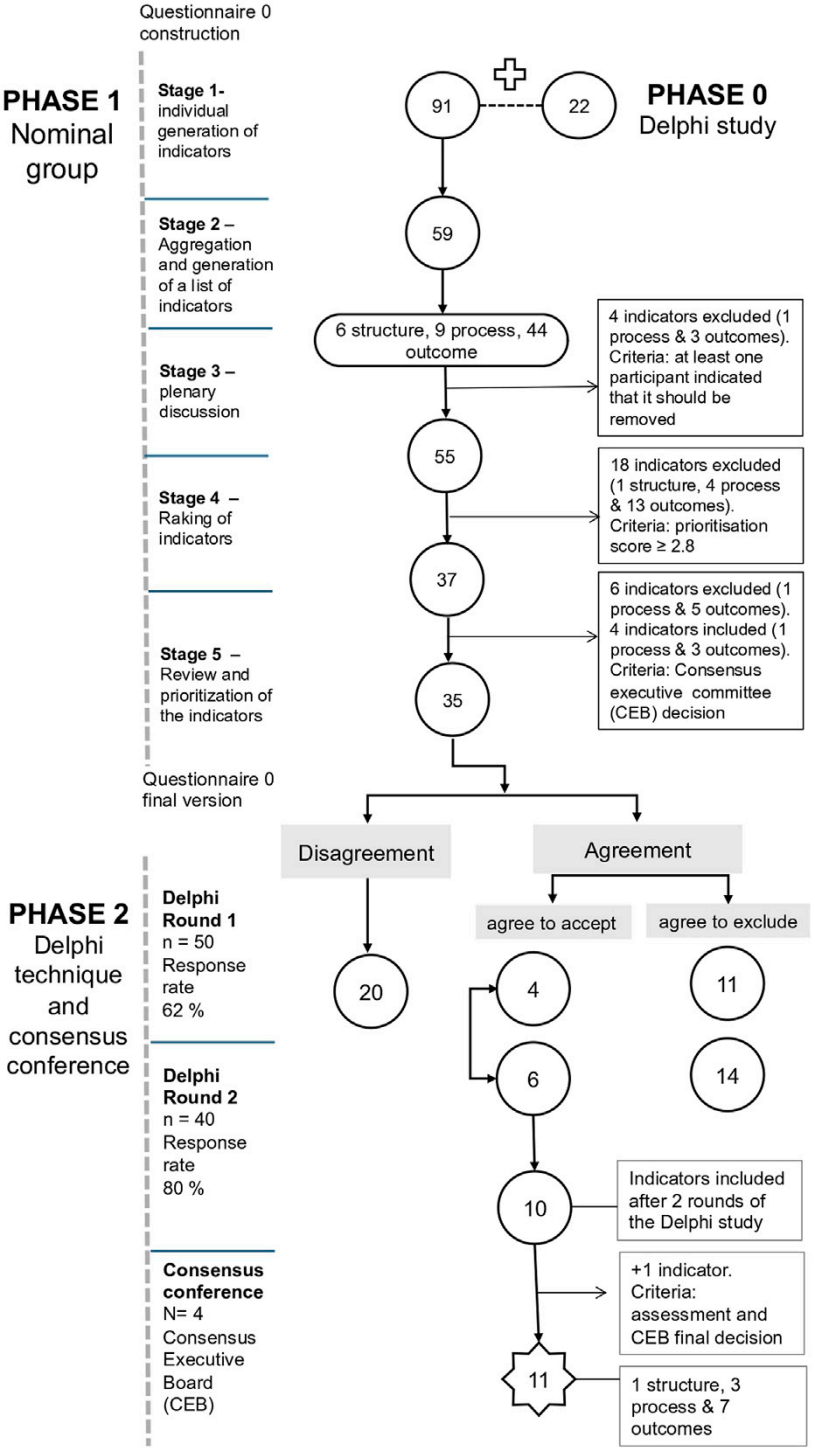
Diagram of the multiphase study (online and Lisbon, Portugal, 2024).

Given that in this study the structural indicators received lower scores comparing with other indicators, the cutoff point was adjusted to ≥2.8 to ensure the inclusion of a comprehensive range of indicators in the subsequent phase of the study (Delphi rounds). Furthermore, six indicators were excluded after deliberation by the CEB due to their lack of clarity and robustness for evaluation in phase 2. A summary of the results from the various stages of the nominal group is presented in Figure 4.

The final rating of the 35 indicators can be consulted in Supplementary Table 6.

### Phase 2

#### First Round

In total, 81 participants were contacted to answer the first round of the Delphi technique. Out of these, 50 participants responded in the first round, resulting in a response rate of 61%. The profile of the participants can be consulted in Supplementary Table 7.

In the first round of the Delphi Study, the 35 indicators identified in the nominal group were evaluated based of the two main criteria: feasibility and sensitivity.

After the first round, four indicators had mean scores of ≥4.0 for both feasibility and sensitivity parameters. The included indicators were directly selected to be evaluated in the consensus conference. Conversely, 11 indicators were excluded based on the mean score of the responses ≤3.5 in at least one of the criteria (feasibility or sensitivity), which mean that in at least on the criteria there not considered to be feasible or sensible. In total, 20 indicators were selected to be evaluated in second round.

#### Second Round

In the second round,a response rate of 80% was achieved (40/50 initial participants).

In the second round, 20 indicators were scored. In this round, 6 indicators were prioritized according with the inclusion criteria defined to this round. Although two indicators were not considered a priority by more than 50% of the participants, these indicators were rated over 4 by over 70% of participants in both feasibility and sensitivity. In this sense we have included them for discussion in the consensus conference.

The excluded indicators across the different phases of the study can be consulted in Supplementary Table 8.

### Final Group of Indicators

The results of the two rounds were summarized by the researchers and assessed for consensus across the expert group. In total, 10 indicators were selected for discussion in the consensus conference from the round 1 and 2.

Additionally, one indicator was included in the final list of indicators. Although this indicator was not prioritized by more than 50% of the participants in the second round neither received score ≥4.0 in the first round, it presented good feasibility and sensitivity rates.

A total of 11 indicators reached consensus across the CEB members. Given that the Delphi technique is an exploratory qualitative research, initially the questionnaire 0 was organized in 4 main domains (as detailed in Supplementary Table 6). However, based on the panel’s evaluation and prioritization of the indicators, the CEB members reclassified these domains according with the new results as follows: awareness and activation of the SV support program, process and structures of SV support programs, impact of SV support program.

The final diagram of the three phases can be consulted in Figure 4.

The final scoreboard of indicators is available in Table 1.

**TABLE 1:** Final consensus indicators for assessing second victim support programs (online, 2024).

Indicators	Type of indicator	Total number of respondents	Feasibility Rated over 4 (%)	Sensitivity Rated over 4 (%)
Awareness and activation of the second victim support program				
1. Number of provided support/number of activation requests	Outcome	50	98.0	70.0
2. Number of provided support/Number of SV^a^ identified from the reporting system ^new^	Outcome	40	100.0	100.0
3. Number of HCWs aware of the SV program/Total number of HCWs^b^	Outcome	40	100.0	100.0
Process and Structures of second victim support Programs				
4. Average time elapsed from the incident to the first encounter ^new^	Process	50	75.0	68.8
5. Existence of a policy strategy for SV support approved by the institution ^new^	Structure	40	92.5	97.5
6. Number of peers supporters receiving training or trained/Total of peer supporters integrating the program	Process	40	100.0	100.0
7. Number of HCWs receiving training on the SV topic/Total of HCWs of the service/unit/institution ^new^	Process	50	88.0	74.0
Impact of second victim support program				
8. Level of psychological distress before and after the program	Outcome	40	95.0	95.0
9. SV’s perceived benefit after the encounter with the peer supporter	Outcome	40	100	100.0
10. SV experience (after attending the program) – qualitative feedback	Outcome	50	78.0	82.0
11. Number of working days lost due to emotional distress in HCWs that attended the SV program/total number of working days lost due to emotional distress ^new^	Outcome	40	97.5	97.5

new–new outcome generated from the study (to the best of our knowledge, these indicator was not used to evaluate other SV, intervention).

^a^ SV, second victim.

^b^ HCW, healthcare worker.

More detailed information about the rating of the indicators is provided in Supplementary File 9. For further information on the description of each indicator, please consult Supplementary File 10. For a detailed explanation of the purpose and measurement method for each indicator, consult Supplementary File 11.

## Discussion

In this study, a multiphase approach was employed to develop a scoreboard of indicators for evaluating SV programs in healthcare institutions. The agreed-upon set of indicators represents an initial effort to establish a common group of feasible and sensitive/responsive metrics for evaluating SV support programs, facilitating the comparison of results over-time and across various contexts. These indicators are critical success factors for the design of SV support programs. Indirectly, these metrics also provide a useful assessment of the level of psychological safety within the institution, gauging the openness of interprofessional risk-taking of seeking support, and evaluating institution readiness to provide necessary training, structures, and process for SV support systems [22, 45–47].

In the design and development of the final 11 indicators, we focused on the scientific rigor recommended by the literature [48], aiming to define measures that are meaningful, generalizable, and interpretable [48].

We have found that seven out of eleven final proposed indicators were already utilized in existing SV programs. This not only reinforces the adequacy of the suggested indicators, but also confirms their applicability and alignment with the evidence-based practices, emphasizing the need to support providers [49]. Another strong point was the high-level participation in the Delphi rounds which included multi-professional and international perspective, incorporating professionals who work directly or indirectly on the SVP, and representing diverse organizational cultures and healthcare system. This increased the robustness and comprehensiveness of the obtained results. Moreover, the expertise and diversity of the participants contributed to the credibility and validity of the data.

In this study we found that outcome indicators are the most valued for SV program evaluation, however we highlight the importance of considering the structural and process indicators to provide a comprehensive view of the program’s effectiveness and ensure that all relevant aspects of the program are being assessed.

We believe that these indicators hold significant potential in guiding program managers towards strategic decisions. These indicators not only can provide key information about programs’ acceptability, but also enlighten about the pivotal factors contributing to the success of these programs. Additionally, all the indicators have low calculation cost which facilitates their practical implementation.

It is crucial to highlight that we prioritized defining a restrict number of indicators that are suitable for various healthcare contexts and levels of implementation and do not overburden health services in terms of time and resources. Our goal is to provide common metrics that enable comparable outcome measures for SV support programs. This approach sets a foundation for future research and the potential inclusion of additional structural and process indicators to better support the implementation of SV programs across diverse healthcare settings.

### Limitations

This study had some limitations. It’s important to acknowledge that the findings were generated and evaluated based on a subjective perception of a selected group of invited experts. This subjectivity may limit the generalizability of the findings (also known as transferability), to different settings and the reliability of the data.

Moreover, generation of indicators was restricted to phases 0 and 1 (literature review and nominal group), limiting interaction among participants who joined during the Delphi rounds. To mitigate these limitations, we employed more adaptable criteria early in the study to expand the range of possibilities for subsequent Delphi rounds, facilitating a broader consensus among participants involved in both the nominal group and Delphi techniques.

According with the multidimensional model of Seys et al, there 5 levels of support that can be provided to the second victims [50]. The final group of defined indicators primarily applies to levels 3 to 5 of this multilevel approach [50], which means that only focus on measuring formal support interventions that provide a reaction after adverse events or other distressing situations happen. We recommend that future research will need to reach a consensus on indicators to evaluate levels 1 and 2 (prevention and self-care) areas, where fewer experiences are reported in health organizations. These dimensions include actions such as trying to understand what happened and how to avoid future similar situations, education on the SV topic and promotion of non-punitive responses to error [50].

Additionally, we identified a lack of prioritization of structural outcomes throughout the different stages of the study. This type of indicators received less attention and only the existence of a policy strategy for SV support, approved by the institution, was prioritized by the majority of the participants. Due to their intangible and less quantifiable nature structural aspects may not receive the same level of attention and accountability, which could diminish the incentive for prioritizing structural enhancements. On the other hand, these indicators are crucial in all stages of implementation, including planning and ongoing adjustments over time [51].

An important aspect missing from this study is the inclusion of indicators that measure the long-term impact and sustainability of SV support programs. We strongly recommend employing a rigorous method to identify the most suitable indicators for assessing this type of programs over time.

### Conclusion

This study has successfully delineated a comprehensive set of 11 indicators crucial for evaluating SV support programs within healthcare services. Achieved through a rigorous consensus method, this scoreboard of indicators integrates both evidence-based findings and empirical insights from a multidisciplinary panel of international experts.

To our knowledge, this is the first study to identify a set of indicators applicable across different healthcare contexts and different settings. Moreover, the applicability of these findings extends beyond healthcare facilities and can be generalized to other institutions that provide care.

This study aims to guide future SV programs by enhancing decision-making in key areas: awareness and activation of the SV program, structural and process improvements, and the impact of these programs. There is, however, a need for further research to establish consensus on indicators for evaluating self-care and prevention strategies in healthcare, particularly in areas where initiatives are currently sparse.

By establishing this common set of indicators, we encourage future research to enrich and expand the application of structural and process indicators, which will enhance the implementation and effectiveness of future SV programs across various healthcare settings.
