# Corrigendum: Developing Core Indicators for Evaluating Second Victim Programs: An International Consensus Approach

**DOI:** 10.3389/ijph.2024.1608061

**Published:** 2024-11-07

**Authors:** Sofia Guerra-Paiva, Irene Carrillo, José Mira, Joana Fernandes, Reinhard Strametz, Eva Gil-Hernández, Paulo Sousa

**Affiliations:** ^1^ Public Health Research Centre, NOVA National School of Public Health, NOVA University of Lisbon, Lisboa, Portugal; ^2^ Comprehensive Health Research Center, NOVA National School of Public Health, NOVA University of Lisbon, Lisboa, Portugal; ^3^ Department of Health Psychology, Miguel Hernández University, Elche, Spain; ^4^ Fundación para el Fomento de la Investigación Sanitaria y Biomédica de la Comunitat Valenciana (FISABIO), Valencia, Spain; ^5^ NOVA National School of Public Health, NOVA University of Lisbon, Lisbon, Lisboa, Portugal; ^6^ Wiesbaden Business School, RheinMain University of Applied Sciences, Wiesbaden, Germany

**Keywords:** patient safety, second victim, programs, evaluation, indicators

There was an error in Affiliation 1. Instead of “NOVA University of Lisbon, Public Health Research Centre, Lisboa, Portugal” it should be “Public Health Research Centre, NOVA National School of Public Health, NOVA University of Lisbon, Lisboa, Portugal.”

There was an error in Affiliation 2. Instead of “Comprehensive Health Research Center, CHRC, NOVA University of Lisbon, Lisboa, Portugal” it should be “Comprehensive Health Research Center, NOVA National School of Public Health, NOVA University of Lisbon, Lisboa, Portugal.”

There was an error in Affiliation 5. Instead of “National School of Public Health, NOVA University of Lisbon, Lisboa, Portugal” it should be “NOVA National School of Public Health, NOVA University of Lisbon, Lisbon, Lisboa, Portugal”.

In the first published version of the article there was an error in the **Acknowledgements** section. The section previously stated:

“This work was supported by ERNST COST Action 19113 (COST–European Cooperation in Science and Technology, www.cost.eu). We extend our acknowledgments to the members of ERNST and invited experts who generously participated in this study.”

This has changed to:

“This article is based upon work from COST Action, The ERNST Group (The European Researchers’ Network Working on Second Victims), CA19113, supported by COST (European Cooperation in Science and Technology). We extend our acknowledgments to the members of ERNST and invited experts who generously participated in this study.”

The authors apologize for these errors and state that this does not change the scientific conclusions of the article in any way. The first published incorrect version of the article has been updated.

