# Supplementary material for: Developing Core Indicators for Evaluating Second Victim Programs: An International Consensus Approach
Source: Int J Public Health. 2024 Aug 30;69:1607428. doi: 10.3389/ijph.2024.1607428 (PMC11392755; doi:10.3389/ijph.2024.1607428)
Supplement: Supplementary file 1 [file DataSheet1.docx]

**International Journal of Public Health**

**Title: Developing Core Indicators for Evaluating Second Victim Programs: An International Consensus Approach**

Supplementary file 1 –Search strategy used in the scoping review following the Context, content and population (Key factors for effective implementation of healthcare workers support interventions after patient safety incidents in health organisations: a scoping review, Lisbon, Portugal, 2023)

| Context #1 | “Health Services” OR “Health Facilities” OR “Healthcare” OR “Primary health care” OR “General Practice” OR “Family practice” OR “Ambulatory Care” OR “Nursing Care” OR “Family unit” OR “Hospitals” |
| --- | --- |
| Content #2 | “Program evaluation” OR “Support program” OR “Peer support” OR “Support strategies” OR “Organizational factors” OR “Organizational culture” |
| Population #3 | (“Health personnel” OR “Physicians” OR “Nurses” OR “Doctor” OR “Practitioner” OR “Medical students” OR “Medical residents” OR “Healthcare providers” OR “Healthcare worker” OR “Healthcare staff”) AND (“Error” OR “Near miss” OR “Adverse Event” OR “Clinical Error” OR “Medical error” OR “Second victim” OR “Wounded caregiver” OR “Wounded healer” OR “Secondary trauma”) |
| Search strategy | #1AND #2 AND #3 |
| Language | No language filter/restraint will be applied |
| Period | no period filter/restraint will be applied |
| Exclusion criteria | Article types not included: editorial, letter to the editor, cases series, case reports, narrative review, commentary |

Supplementary file 2- Flowchart of the scoping review “Key factors for effective implementation of healthcare workers support interventions after patient safety incidents in health organisations: a scoping review” . doi:10.1136/ bmjopen-2023-078118 (Key factors for effective implementation of healthcare workers support interventions after patient safety incidents in health organisations: a scoping review, Lisbon, Portugal, 2023)

Records identified from databases*:

(n= 9708)

*CINAHL (n=1163)

*Cochrane Library (n=0)

*Embase ‎ (n=684)

*Epistemonikos ‎(n=106)

*Psycinfo ‎ (n=787)

*PubMed ‎ (n=743)

*SciELO Citation Index (n=146)

*Scopus ‎ (n=5074)

*Web of Science Core Collection (n=1005)

Total evaluated in Rayyan - 6477

Total evaluated in Rayyan (updated) –9189 COM DUPLICADOS

**Identification of studies via other methods**

**Identification of studies via databases and registers**

Records identified from:

Websites (n=11)

Citation searching (n=43)

Stakeholders group (n=4)

Duplicate records removed *before screening*:

EndNote (n= 465)

Rayyan (n= 1863)

**Identification**

Reports not retrieved

(n = 1 website + 6 references)

Reports sought for retrieval

(n=58)

Records screened (n=7370)

Records excluded by humans

(n= 7259)

Reports excluded (n=39):

- There is no information if the intervention was successfuly implemented (n=6)
- Absence of data related with implementation process (n =4)
- Did not describe a specific type of support intervention / course (n=24)
- Different population focus-The intervention is not related with supporting healthcare workers (n=4)
- The collected information didn’t filled in more than 6 categories of our collating data framework (n=1)

**Screening**

Reports not retrieved

(n = 6)

Reports sought for retrieval

(n=111)

Reports assessed for eligibility

(n=51)

Reports excluded (n=88):

- There is no clear information if the intervention was successfuly implemented (n=12 )
- Absence of data related with implementation process (n=9)
- Results already presented in other article/document (n=4)
- Did not describe a specific type of support intervention (n=60)
- Different population focus-The intervention is not related with supporting healthcare workers(n=2)
- Does not complete 7 domains of the collating the data framework (n=1)

Reports assessed for eligibility

(n=105)

Studies included in the review

(n=29)

| **SECOND VICTIM EXPERIENCE AFTER ATTENDING THE PROGRAM**   - Overall experience of the participants after attending the intervention (relevance and usefulness of the programme^1-13^ |
| --- |
| **HEALTH OUTCOMES**   - Emotional, psychological and physical distress^14;15^ - Work-related symptoms ^10;11^ - General health and vitality; Emotion regulation; Perception of stress, anxiety and burnout^13; 15;16;17;18^ - Quality of life assessment ^2^ |
| **STAFF COMPETENCES AFTER ATTENDING THE PROGRAMME**   - Staff awareness of second victim phenomenon ^16^ - Sense of preparedness in handling critical events and confidence in coping with adverse events and knowledge about resilience and coping strategies ^13^ - Knowledge improvement (main outcome) ^1,19^ |
| **WORK RELATED OUTCOMES**   - Job satisfaction^2^ - Turnover intentions ^15^ |
| **SELF PERCEPTIONS**   - Perceived helpfulness of the peer support group program ^14;20^ - Feeling cared by the institution  ^21^ |
| **IMPACT ON HEALTHCARE ORGANIZATION**   - Impact on culture of the department  ^21^ |
| **PROGRAM OUTCOMES**   - Attendance to the sessions ^2, 5,13,16,19,23^ - Frequency of program activation ^24,3,5,23, 6,7,8,9,15,25,26,27,28^ - Interactions resolutions (number of interactions solved in real time) 26, 20, 29 - No of HCWs who need external support 29 |
| **STRUCTURE OUTCOMES**   - The existence of formalized structures directed at fostering a PS culture, based on a just culture approach ^3-5,16,19,30^ - Active involvement of leadership members in initiatives that support SVs and HCWs’ well-being ^3,16, 23, 7, 14 , 15, 25^ |
| **PROCESS OUTCOMES**   - Training of second victim peer supporters ^2, 3, 5, 6, 7,9-11, 15, 16, 23, 25- 27, 29, 31^ - Dissemination was also carried out for recruiting peer supporters ^,6, 7, 23, 25^ - Involvement of the leadership members in the dissemination process ^9, 31^ |

Supplementary file 3 – Indicators selected in the scoping review. (Key factors for effective implementation of healthcare workers support interventions after patient safety incidents in health organisations: a scoping review, Lisbon, Portugal, 2023)

1. Mira JJ, Carrillo I, Guilabert M, *et al*. The second victim phenomenon after a clinical error: the design and evaluation of a Website to reduce Caregivers’ emotional responses after a clinical error. *J Med Internet Res* 2017;19:e203.

2. Graham P , Zerbi G, Norcross W, *et al*. Testing of A Caregiver support team. *Explore (NY)* 2019;15:19–26.

1. Schuster MA. Creating the hematology/oncology/stem cell transplant advancing resiliency team: A nurse-led support program for hematology/oncology/stem cell transplant staff. *J Pediatr Oncol Nurs* 2021;38:331–41.
2. Foreman S. Developing a process to support perinatal nurses after a critical event. *Nurs Womens Health* 2014;18:61–5.
3. Civil NM, Hoskins JD. Building a critical incident peer response team: A full theatre team welfare intervention. *Anaesth Intensive Care* 2022;50:421–9.
4. Krzan KD, Merandi J, Morvay S, *et al*. “Implementation of a “second victim” program in a pediatric hospital”. *Am J Health Syst Pharm* 2015;72:563–7.
5. Finney RE, Jacob A, Johnson J, *et al*. Implementation of a second victim peer support program in a large anesthesia Department. *AANA* 2021;89:235–44.
6. Schrøder K, Bovil T, Jørgensen JS, *et al*. Evaluation Of’The buddy study’, a peer support program for second victims in Healthcare: a survey in two Danish hospital departments. *BMC Health Serv Res* 2022;22:566.
7. Merandi J, Winning AM, Liao N, *et al*. Implementation of a second victim program in the neonatal intensive care unit: an interim analysis of employee satisfaction. *Journal of Patient Safety and Risk Management* 2018;23:231–8.
8. Calder-Sprackman S, Kumar T, Gerin-Lajoie C, *et al*. Ice cream rounds: the adaptation, implementation, and evaluation of a peer- support wellness rounds in an emergency medicine resident training program. *CJEM* 2018;20:777–80.
9. Peterson U, Bergström G, Samuelsson M, *et al*. Reflecting peer- support groups in the prevention of stress and burnout: randomized controlled trial. *J Adv Nurs* 2008;63:506–16.

Allen D, Spencer G, McEwan K, *et al*. The Schwartz centre rounds: supporting mental health workers with the emotional impact of their work. *Int J Ment Health Nurs* 2020;29:942–52.

1. Johnson J, Simms-Ellis R, Janes G, *et al*. Can we prepare Healthcare professionals and students for involvement in stressful Healthcare events? A mixed-methods evaluation of a resilience training intervention. *BMC Health Serv Res* 2020;20:1094.
2. Mellins CA, Mayer LES, Glasofer DR, *et al*. Supporting the well- being of health care providers during the COVID-19 pandemic: the Copecolumbia response. *Gen Hosp Psychiatry* 2020;67:62–9.
3. Thompson M, Hunnicutt R, Broadhead M, *et al*. Implementation of a certified registered nurse Anesthetist second victim peer support program. *J Perianesth Nurs* 2022;37:167–73.
4. Bryant R. *Raising Awareness of Second Victim Phenomenon and SupportingYOU: A Quality and Safety Initiative. Graduate Theses, Dissertations, and Problem Reports*. West Virginia: West Verginia University, 2022: 10169.
5. Bernburg M, Groneberg DA, Mache S. Mental health promotion intervention for nurses working in German psychiatric
   hospital departments: A pilot study. *Issues Ment Health Nurs* 2019;40:706–11.
6. Hinzmann D, Forster A, Koll-Krüsmann M, *et al*. Calling for help- peer-based Psychosocial support for medical staff by telephone-A best practice example from Germany. *Int J Environ Res Public Health* 2022;19:15453.
7. Rubin EW, Rassman A. cAIR: implementation of peer response support for frontline health care workers facing the COVID-19 pandemic. *Soc Work Health Care* 2021;60:177–86.
8. Rivera-Chiauzzi EY, Smith HA, Moore-Murray T, et al. Healing our own: A randomized trial to assess benefits of peer support. J Patient Saf 2022;18:e308–14.
9. Sexton JB, Adair KC, Profit J, Milne J, McCulloh M, Scott S, et al. Perceptions of Institutional Support for “Second Victims” Are Associated with Safety Culture and Workforce Well-Being. Jt Comm J Qual Patient Saf. 2021 May;47(5):306–12.
10. Quillivan RR, et al. Patient safety culture and the second victim phenomenon: connecting culture to staff distress in nurses. Jt Comm J Qual Patient Saf. 2016;42:377–386.
11. Shapiro J, Galowitz P. Peer support for Clinicians: A programmatic approach. *Acad Med* 2016;91:1200–4.
12. Cobos-Vargas A, Pérez-Pérez P, Núñez-Núñez M, *et al*. Second victim support at the core of severe adverse event investigation. *Int J Environ Res Public Health* 2022;19:16850.
13. El Hechi MW, Bohnen JD, Westfal M, *et al*. Design and impact of a novel surgery-specific second victim peer support program. *J Am Coll Surg* 2020;230:926–33.
14. Lane MA, Newman BM, Taylor MZ, *et al*. Supporting Clinicians after adverse events: development of a clinician peer support program. *J Patient Saf* 2018;14:e56–60.
15. Merandi J, Liao N, Lewe D, *et al*. Deployment of a second victim peer support program: A replication study. *Pediatr Qual Saf* 2017;2:e031.
16. Morales CL, Brown MM. Creating a care for the Caregiver program in a ten-hospital health system. *Crit Care Nurs Clin North Am* 2019;31:461–73.
17. Scott SD, Hirschinger LE, Cox KR, *et al*. Caring for our own: deploying a systemwide second victim rapid response team. *Jt Comm J Qual Patient Saf* 2010;36:233–40.
18. Roesler R, Ward D, Short M. Supporting Staff Recovery and Reintegration After a Critical Incident Resulting in Infant Death. Adv Neonatal Care. 2009;9(4):163–71.
19. Edrees H, Connors C, Paine L, Norvell M, Taylor H, Wu AW. Implementing the RISE second victim support programme at the Johns Hopkins Hospital: a case study. BMJ Open. 2016;6(9):e011708.

Supplementary file 4 - Demographic characterization of the participants of the nominal group (Lisbon, Portugal, 2024)

| **Country** | **N** |
| --- | --- |
| Croatia | 2 |
| Slovakia | 1 |
| Portugal | 1 |
| Spain | 2 |
| Estonia | 1 |
| Germany | 1 |
| Malta | 1 |
| Poland | 1 |
| Israel | 1 |
| Norway | 2 |
| Finland | 1 |
| Serbia | 1 |
| **Professional Background** |  |
| Psychology | 2 |
| Medicine | 9 |
| Pharmacology | 1 |
| Nurse | 2 |
| Business Economics and Management | 1 |
| Sex |  |
| Female | 9 |
| Male | 6 |
| Total | 15 |

Supplementary file 5- rating of the indicators in the nominal group (online, 2024)

| **INDICATOR** | **Mean (1-5)** | **CV** |
| --- | --- | --- |
| 1. Number of HCWs receiving training on the SV topic/Total of HCWs | 4.4 | 0.2 |
| 2. Number of provided support/number of activation requests | 4.1 | 0.2 |
| 3. SV experience (after attending the program) - qualitative feedback / Likert scale… | 3.9 | 0.3 |
| 4. Average time elapsed from the incident to the first encounter | 3.8 | 0.3 |
| 5. Number of peers supporters receiving training or trained/Total of peer supporters | 3.8 | 0.3 |
| 6. % of professional that increase job satisfaction after and before attending support program | 3.6 | 0.3 |
| 7. SV's perceived benefit after the encounter with the peer supporter | 3.6 | 0.4 |
| 8. Number of support interventions^3^ | 3.5 | 0.2 |
| 9. Number of HCWs aware of the SV program/total number of HCWs | 3.5 | 0.3 |
| 10. Program recommendations from the users | 3.5 | 0.3 |
| 11. Number of HCWs involved in the organization of the support program^5^ | 3.5 | 0.3 |
| 12. Total of SV supported/Number of SV identified from the reporting system | 3.4 | 0.3 |
| 13. Existence of a training protocol for peer supporters | 3.4 | 0.3 |
| 14. HCWs who attended the program and feel cared by the institution/ total number of HCWs that attended the program^4^ | 3.4 | 0.3 |
| 15. Level of psychological distress before and after the program | 3.4 | 0.3 |
| 16. Number of referrals for specialized care | 3.4 | 0.4 |
| 17. Number of HCWs aware of the SVP/total number of HCWs | 3.4 | 0.4 |
| 18. HCWs capable of speaking up after the occurrence of an adverse event/HCWs involved in the adverse event^4^ | 3.4 | 0.4 |
| 19. HCWs perception of resilience before and after attending the support program | 3.4 | 0.3 |
| 20. Quality of life before and after attending the support program^4^ | 3.4 | 0.4 |
| 21. Number of HCWs who got 1st level of peer support (informal peer support)^7^ | 3.4 | 0.4 |
| 22. Existence of a policy strategy for SVs approved by the institution | 3.4 | 0.4 |
| 23. Overall perceived benefit of the program from the staff organization | 3.3 | 0.4 |
| 24. Feeling of safety in the aftermath of an adverse event | 3.3 | 0.5 |
| 25. Number of peers recruited for the support program | 3.2 | 0.3 |
| 26. Levels of acute stress following an incident before and after the program | 3.2 | 0.4 |
| 27. Number of sentinel events/ total of adverse events registered in the organization^3^ | 3.1 | 0.4 |
| 28. Peer supporter’s perceived benefit after the encounter^4^ | 3.1 | 0.4 |
| 29. SV with psychological sequels after 1 year/HCWs that attended the support program | 3.1 | 0.4 |
| 30. SV intention to leave job after attending the support program/total number of SV that attended the support program | 3.1 | 0.4 |
| 31. Patients adequately informed after the adverse event/ total number of patients involved in adverse events^8^ | 3.0 | 0.4 |
| 32. Perception of patient safety culture before and after attending the support program | 3.0 | 0.4 |
| 33. Number of peer supporters team meetings per year^9^ | 2.9 | 0.4 |
| 34. Number of HCWs supported without signs of SV/total number of supported HCWs after healthcare incidents | 2.9 | 0.4 |
| 35. Existence of a monitoring committee | 2.9 | 0.5 |
| 36. HCWs that were involved in another adverse event after attending the program/total number of HCWs that attended the program^3^ | 2.8 | 0.4 |
| 37. Duration of peer support encounters^4^ | 2.8 | 0.4 |
| 38. Number of patient complaints after adverse events | 2.8 | 0.4 |
| 39. Number of cases attended by gender, profile, type of incident^3^ | 2.8 | 0.5 |
| 40. Number of meetings between the support team leader and the board of the institution. | 2.8 | 0.5 |
| 41. Monetary losses after an adverse event in hospital /total costs of implementing the program^1^ | 2.8 | 0.5 |
| 42. Sense of preparedness in handling stressful incidents^4^ | 2.7 | 0.4 |
| 43. Number of suicides after attending the support program/Total number of HCWs that attended the support program^2^ | 2.7 | 0.5 |
| 44. Number of HCWs willing to support others after adverse events/total number of HCWs^2^ | 2.7 | 0.5 |
| 45. Average number of interactions necessary for including the HCW in the program after an incident | 2.6 | 0.4 |
| 46. Number of HCWs aware of legal procedures to follow after an adverse event/ Total of HCWs | 2.6 | 0.4 |
| 47. SV with physical consequences after 1 year/HCWs that attended the support program | 2.6 | 0.4 |
| 48. Average number of working days lost because emotional distress^2^ | 2.6 | 0.5 |
| 49. Modality of the intervention of the support program | 2.5 | 0.4 |
| 50. Existence of return-to-work procedure after sick leave | 2.4 | 0.3 |
| 51. Number of protected working hours for activities related with the support program | 2.4 | 0.4 |
| 52. Number of HCWs being transferred to different departments/ Number of total HCWs that attended the program | 2.4 | 0.5 |
| 53. Ongoing litigation (on an organizational level) | 2.4 | 0.5 |
| 54. Average number of interactions necessary for supporting the HCWs after a healthcare incident ^2, 6^ | 2.3 | 0.5 |
| 55. Patient satisfaction | 2.1 | 0.6 |
| 56. Number of rejected cases (negligence, malpractice) | 1.9 | 0.4 |
| 57. Date of the last evaluation or update of the intervention | 1.9 | 0.5 |
| 58. Peer supporters’ profile | 1.7 | 0.5 |
| 59. Provider satisfaction (managers, HCWs, Healthcare institution) | 1.7 | 0.6 |

SV – second victim ; HCW – healthcare worker; SVP – Second Victim Phenomenon

1.Applied a cutoff for inclusion where indicators scored ≥ 2.8; 2. Included metrics to be evaluated in the second phase of the study (Delphi technique); 3. Metrics were excluded if at least one participant indicated that they should be removed; 4. Excluded indicators by the Consensus Committee; 5. The indicator was adjusted from “Number of HCWs involved …” to “Ratio of HCWs involved…” 6. After discussion of the Consensus Committee, this indicator was adjusted to “Mean of peer encounters”; 7. The indicator was adjusted from “Number of HCWs who got 1st level” to “Ratio of HCWs…”; 8. The indicators was adjusted from “Patient adequately informed after…” to “Ratio of patients adequately informed…”; 9. The indicator was adjusted from “number of peer supporters team meetings” to “ratio of peer supporters team”.

Supplementary file 6- rating of the indicators in the nominal group according with the 4 categories (online, 2023)

| **Indicators related to the second victim program** |
| --- |
| 1. Number of provided support/number of activation requests |
| 2. Total of SV supported/Number of SV identified from the reporting system |
| 3. Number of HCWs supported without signs of SV/total number of supported HCWs after healthcare incidents |
| 4. Number of referrals for specialized care |
| 5. Monetary losses after an adverse event in hospital/total costs of implementing the program |
| **Indicators related to the intervention process and structure** |
| 6. Number of peers supporters receiving training or trained/Total of peer supporters |
| 7. Ratio of HCWs involved in the organization of the support program |
| 8. Existence of a training protocol for peer supporters (dichotomic response Yes/No) |
| 9. Existence of a monitoring committee |
| 10. Existence of a policy strategy for SVs approved by the institution |
| 11. Number of peers recruited for the support program |
| 12. Ratio of peer supporters team meetings per year |
| 13. Mean of peer support encounters^1^ |
| 14. Number of meetings between the support team leader and the board of the institution |
| 15. Average time elapsed from the incident to the first encounter |
| **Indicators related to the second victim experience** |
| 16. SV's perceived benefit after the encounter with the peer supporter |
| 17. SV experience (after attending the program) - qualitative feedback / Likert scale… |
| 18. % of professional that increase job satisfaction after and before attending support program |
| 19. Program recommendations from the users |
| 20. Level of psychological distress before and after the program |
| 21. HCWs perception of resilience before and after attending the support program |
| 22. Ratio of HCWs who got 1st level of peer support (informal peer support) |
| 23. Feeling of safety in the aftermath of an adverse event |
| 24. Levels of acute stress following an incident before and after the program |
| 25. SV with psychological sequels after 1 year/total number of SV that attended the support program |
| 26. SV intention to leave job after attending the support program/total number of SV that attended the support program |
| 27. Perception of patient safety culture before and after attending the support program |
| 28. Average number of working days lost because of emotional distress |
| 29. Number of suicides after attending the support program/Total number of HCWs that attended the support program |
| **Indicators related to the healthcare organization and culture** |
| 30. Number of HCWs aware of the SV program/total number of HCWs |
| 31. Number of HCWs receiving training on the SV topic/Total of HCWs |
| 32. Number of HCWs willing to support others after adverse events/total number of HCWs |
| 33. Overall perceived benefit of the program from the staff organization |
| 34. Ratio of patients adequately informed after the adverse event / total number of patients involved in adverse events |
| 35. Number of patient complaints after adverse events |

1.After discussion of the Consensus Committee, this indicator was adjusted from the indicator “Average number of interactions necessary for supporting the HCWs after a healthcare incident”;

SV – second victim ; HCW – healthcare worker;

Supplementary file 7- Demographic characterization of the participants that joined the Delphi Technique (online, 2024)

| **Country** | **N** |
| --- | --- |
| Argentina | 1 |
| Belgium | 2 |
| Bosnia and Herzegovina | 2 |
| Brasil | 1 |
| Chile | 1 |
| Croatia | 2 |
| Estonia | 2 |
| Finland | 1 |
| Germany | 2 |
| Iceland | 2 |
| Ireland | 2 |
| Israel | 1 |
| Italia | 2 |
| Macedonia | 2 |
| Malta | 1 |
| Netherland | 1 |
| Norway | 2 |
| Poland | 1 |
| Portugal | 2 |
| Romenia | 1 |
| Serbia | 4 |
| Slovakia | 2 |
| Spain | 5 |
| Sweden | 1 |
| Turkey | 4 |
| Ukrania | 1 |
| USA | 1 |
| France | 1 |
| **Professional Background** |  |
| Economics and Management | 2 |
| Medicine | 27 |
| Nursing | 8 |
| Pharrmacy | 5 |
| Psychology | 8 |
| **Sex** |  |
| Female | 35 |
| Male | 15 |
| **Total** | 50 |

Supplementary file 8 - Excluded indicators (online, 2024)

| **PHASE 1 – nominal group (59 indicators to 35 indicators- 24 excluded indicators)** |
| --- |
| Number of support interventions |
| Number of HCWs aware of the SVP/total number of HCWs |
| HCWs who attended the program and feel cared by the institution/ total number of HCWs that attended the program |
| HCWs capable of speaking up after the occurrence of an adverse event /HCWs involved in the adverse event |
| Quality of life before and after attending the support program |
| Number of sentinel events/ total of adverse events registered in the organization |
| Peer supporter’s perceived benefit after the encounter |
| HCWs that were involved in another adverse event after attending the program /total number of HCWs that attended the program |
| Duration of peer support encounters |
| Number of cases attended by gender, profile, type of incident |
| Sense of preparedness in handling stressful incidents |
| Average number of interactions necessary for including the HCW in the program after a healthcare incident |
| Number of HCWs aware of legal procedures to follow after an adverse event/ Total of HCWs |
| SVs with physical consequences after 1 year/HCWs that attended the support program |
| Modality of the intervention of the support program |
| Existence of return-to-work procedure after sick leave |
| Number of protected working hours for activities related with the support program |
| Number of HCWs being transferred to different departments/Number of total HCWs that attended the program |
| Ongoing litigation (on an organizational level) |
| Patient satisfaction |
| Number of rejected cases (negligence, malpractice) |
| Date of the last evaluation or update of the intervention |
| Peer supporters’ profile |
| Provider satisfaction (managers, HCWs, Healthcare institution) |
| **DELPHI ROUND – 1^ST^ WAVE (11 indicators excluded)** |
| Number of HCWs supported without signs of SV/total number of supported HCWs after healthcare incidents |
| Monetary losses after an adverse event in hospital/total costs of implementing the program |
| Existence of a monitoring committee |
| Mean of peer support encounters |
| Number of meetings between the support team leader and the board of the institution |
| HCWs perception of resilience before and after attending the support program |
| Ratio of HCWs who got 1st level of peer support (informal peer support) |
| Feeling of safety in the aftermath of a healthcare incident |
| Levels of acute stress following a healthcare incident before and after the program |
| SV with psychological sequels after 1 year/total number of SV that attended the support program |
| Ratio of patients adequately informed after the adverse event / total number of patients involved in adverse events |
| **DELPHI ROUND - 2^ND^ WAVE (14 indicators excluded )** |
| Number of referrals for specialized care |
| Ratio of HCWs involved in the organization of the support program |
| Existence of a training protocol for peer supporters (dichotomic response Yes/No) |
| Number of peers recruited for the support program |
| Ratio of peer supporters team meetings per year |
| Number of HCWs willing to support others after adverse events/total number of HCWs**^1^** |
| % of professional that increase job satisfaction after and before attending support program |
| Program recommendations from the users |
| SV intention to leave job after attending the support program/total number of SV that attended the support program |
| Perception of patient safety culture before and after attending the support program |
| Average number of working days lost because of emotional distress^1^ |
| Number of suicides after attending the support program/Total number of HCWs that attended the support program |
| Overall perceived benefit of the program from the staff organization |
| Number of patient complaints after adverse events |

1.Indicator that was included for discussion in the consensus conference based on CEB decision; SV – second victim ; HCW – healthcare worker;

Supplementary file 9- Evaluation of the final list of indicators (online, 2024)

|  | **FEASIBLE** | | | | | | **SENSITIVE** | | | | | |
| --- | --- | --- | --- | --- | --- | --- | --- | --- | --- | --- | --- | --- |
| **Indicators related to the second victim program** | **N** | **M** | **SD** | **CV** | **% ≤ 2** | **% ≥ 4** | **N** | **M** | **SD** | **CV** | **% ≤ 2** | **% ≥ 4** |
| 1. Number of provided support/number of activation requests | 50 | 4,6 | 0,5 | 0,12 | 0.0 | 98,0 | 50 | 4.0 | 1.0 | 0.24 | 8.0 | 70.0 |
| 3. Number of HCWs receiving training on the SV topic/Total of HCWs | 50 | 4.4 | 0.8 | 0.19 | 2.0 | 88.0 | 50 | 4.1 | 1.0 | 0.23 | 4.0 | 74.0 |
| 2. SV experience (after attending the program) - qualitative feedback / Likert scale… | 50 | 4.1 | 0.9 | 0.22 | 6.3 | 78.0 | 50 | 4.2 | 0.8 | 0.19 | 2.0 | 82.0 |
| 4. Average time elapsed from the incident to the first encounter | 50 | 4,0 | 1,0 | 0,24 | 6,3 | 75,0 | 50 | 4,0 | 1,0 | 0,24 | 4,2 | 68,8 |
| 5. Existence of a policy strategy for SVs approved by the institution | 40 | 4.7 | 0.6 | 0.12 | 0.0 | 100 | 40 | 3.7 | 0.9 | 0.24 | 15.0 | 100,0 |
| 6. Number of peers supporters receiving training or trained/  Total of peer supporters | 40 | 4.5 | 0.7 | 0.16 | 2.5 | 100 | 40 | 3.8 | 0.8 | 0.20 | 7.5 | 100,0 |
| 7. Total of SV supported/Number of SV identified  from the reporting system | 40 | 4.1 | 1.1 | 0.27 | 10.0 | 92.5 | 40 | 3.7 | 0.9 | 0.25 | 12.5 | 97.5 |
| 8. Number of HCWs aware of the SV program/  total number of HCWs | 40 | 4.1 | 0.8 | 0.20 | 7.5 | 100 | 40 | 4.0 | 0.6 | 0.15 | 2.5 | 100 |
| 9. SV´s perceived benefit after the encounter with the peer supporter | 40 | 3.7 | 0.7 | 0.18 | 5.0 | 100 | 40 | 3.9 | 0.7 | 0.17 | 0.0 | 100 |
| 10. Number of working days lost due to emotional  distress in HCWs that attended the SV program | 40 | 3.7 | 1.0 | 0.28 | 12.5 | 95.0 | 40 | 3.6 | 1.0 | 0.27 | 12.5 | 95.0 |
| 11. Level of psychological distress before and after the program | 40 | 3.6 | 0.8 | 0.22 | 10.0 | 100 | 40 | 3.6 | 0.8 | 0.23 | 10.0 | 100 |

SV – second victim; HCW – healthcare worker;

Supplementary file 10- Description of the final list of indicators after the application of the consensus methodologies (online, 2024)

| **Awareness and activation of the second victim support program** | |
| --- | --- |
| 1. Number of provided support/ number of activation requests | Number of provided support to SVs after the program activation request /total number of SV program activation requests |
| 2. Number of provided support /Number of SV identified from the reporting system | Number of provided support to SVs after the program activation request/ Total number of HCWs with or without signs of SVP identified from the reporting system |
| 3. Number of HCWs aware of the SV program/ Total number of HCWs | Number of HCWs that refer to now about the existence of the SV program/ Total number of HCWs of the unit/department/ institution (depending of the target of the SV program) |
| **Process and Structures of second victim support Programs** | |
| 4. Average time elapsed from the incident to the first encounter | Average time elapsed from the moment of the adverse event or other type of stressful incidents to the first encounter provided by the SV program |
| 5. Existence of a policy strategy for SVs support approved by the institution | Existence of a policy strategy (formal document, other type of guidelines) for SV support approved by the institution |
| 6. Number of peers supporters receiving training or trained/Total of peer supporters | Number of peers supporters receiving training(or trained) for supporting SVs/ Total of peer supporters integrating the program in the institution |
| 7. Number of HCWs receiving training on the SV topic/Total of HCWs | Number of HCWs from the unit/department/institution depending of the target of the SV program) that integrated a training on the SV topic/ Total of HCWs of the service/unit/institution |
| **Impact of second victim support program** | |
| 8. Level of psychological distress before and after the program | This measure needs to use validated psychological distress scale before and after the SV Program intervention. |
| 9. SV´s perceived benefit after the encounter with the peer supporter | This measure is based on concrete qualitative information of the SV after attending the SV program encounter with the peer supporter.. |
| 10. SV experience (after attending the program) | This measure is based on concrete qualitative information of the SV after attending all the SV program. |
| 11. Number of working days lost due to emotional distress/ total number of working days lost due to emotional distress^1^ | Number of working days lost due to emotional distress (e.g., anxiety, depression, burnout) in HCWs that fully attended the SV program/total number of working days lost due to emotional distress |

1.Additional indicator included by the CEB members in the consensus conference; SV – second victim ; SVP – second victim phenomenon; HCW – healthcare worker

Supplementary file 11 – Purpose and methods of measurement of the final group of indicators after the application of the consensus methodologies (online, 2024)

| **Indicator** | **Purpose** | **Method of measurement** |
| --- | --- | --- |
| 1. Number of provided support/number of activation requests | To identify the number of support requests that were attended after the activation of the program | Consider the program activation requests that were fulfilled with support. The existence of a database to register the program activation requests and the number of provided support is highly recommended. |
| 2. Number of provided support /Number of SV^1^ identified from the reporting system | To identify the number of healthcare workers that experience SV symptoms using the reporting system | The number of SV can be identified using the adverse events reports or other type of reports related with healthcare workers safety . The existence of a database to register the number of provided support is highly recommended. |
| 3. Number of HCWs aware of the SV program/ Total number of HCWs | To determine how many HCWs are aware of the SV program. | A survey can be applied in the service/unit/institution.to determine if HCWs are aware of the SV program. The total number of HCWs should only consider who have received the survey. The survey can be applied online to facilitate the data analysis. |
| 4. Average time elapsed from the incident to the first encounter | To measure the period of time from the program activation to the first encounter | Quantify the period of time from program activation to the first encounter. Calculate the average time. |
| 5. Existence of a policy strategy for SVs support approved by the institution | To ensure that the policy not only exists but is formally approved by the appropriate authorities within the institution. | Identify written documents that outline procedures, rules, or guidelines and are intended to guide actions within the organization. |
| 6. Number of peers supporters receiving training or trained/Total of peer supporters | To ensure that all peers are adequately trained to provide an adequate peer support program | Compile a list of all peer supporters who are part of the program and identify who received training or are receiving training at the moment of the measurement. Obtain the training enrollment records from the training program's administrative office or database |
| 7. Number of HCWs receiving training on the SV topic/Total of HCWs | To determine the extent to which the SVP has been disseminated among healthcare workers | These records should include a list of all HCWs who signed up for and attended the SVP dissemination sessions for HCW. The total number of HCWs of the service/unit/institution can be consulted in the administrative services of the healthcare institution. |
| 8. Level of psychological distress before and after the SV program | To evaluate the impact of the program in the symptoms of psychological distress among the SVs that received support | This can be measured using a validated scale (e.g. Kessler Psychological Distress Scale, K10). The measurement should be done before and after |
| 9. SV´s perceived benefit after the encounter with the peer supporter | To evaluate the degree in which peer supporter program is achieving its intended outcomes and enhance program credibility | Create and apply a survey or questionnaire to gather feedback from individuals who have had encounters with peer supporters. The survey should include questions that capture various dimensions of the perceived benefit (e.g. increased knowledge, emotional support). It’s important to unsure the confidentiality of the data. |
| 10. SV experience (after attending the program) – qualitative feedback | Gain a deeper understanding of participants' experiences and the program's impact on SVs | To apply a survey (open-ended questions) to understand the overall experience of SV’s after attending the program. It’s important to unsure the confidentiality of the data. |
| 11. Number of working days lost due to emotional distress (e.g., anxiety, depression, burnout) in HCWs that fully attended the SV program/total number of working days lost due to emotional distress^1^ | To understand the impact of the program in the reduction of number of working days lost due to emotional distress | Quantify the total number of working days lost due to emotional distress among HCWs who completed the SV program (full completion of the program) . This information can be collected in different sources such as absenteeism records, occupational health records, payroll department, occupational health and safety services. |

1.Additional indicator included by the CEB members in the consensus conference; SV – second victim ; SVP – second victim phenomenon; HCW – healthcare worker
